# Optimization of nitrogen source supply for enhanced biosynthesis and quality of poly(3‐hydroxybutyrate‐*co*‐3‐hydroxyvalerate) by extremely halophilic archaeon *Haloferax mediterranei*

**DOI:** 10.1002/mbo3.1055

**Published:** 2020-05-15

**Authors:** Diya Alsafadi, Othman Al‐Mashaqbeh, Aya Mansour, Majd Alsaad

**Affiliations:** ^1^ Biosynthesis and Biocatalysis Research Unit Foundational Science Research Division Royal Scientific Society Amman Jordan; ^2^ Emerging Pollutants Research Unit Royal Scientific Society Amman Jordan

**Keywords:** carbon‐to‐nitrogen ratio, *Haloferax mediterranei*, poly(3‐hydroxybutyrate‐*co*‐3‐hydroxyvalerate), polyhydroxyalkanoates, yeast extract

## Abstract

The extreme halophilic archaeon, *Haloferax mediterranei* can accumulate polyhydroxyalkanoate (PHA) from different renewable resources. To enhance the biosynthesis and quality of PHA, *H. mediterranei* cultivation media was optimized at different C/N ratios using glucose as the main carbon source. Three sets of media (yeast extract [YE], NH_4_Cl and combination of YE and NH_4_Cl) were prepared at different nitrogen concentrations to achieve C/N ratios of 9, 20, and 35, respectively. The media containing YE (organic nitrogen source) produced a higher growth rate of *H. mediterranei* than NH_4_Cl (inorganic source) at all tested C/N ratios. The highest PHA accumulation (18.4% PHA/cell dry mass) was achieved in a media that combined YE with NH_4_Cl at a C/N ratio of 20. Analysis of the produced polymers revealed the production of poly(3‐hydroxybutyrate‐*co*‐3‐hydroxyvalerate) (PHBHV) with different 3‐hydroxyvalerate (3HV) content. The polymers produced from YE and the combined media have greater 3HV content (10 mol%) than those polymers recovered from NH_4_Cl (1.5 mol%). Resultingly, PHBHV from YE and the combined media displayed reduced melting points at 144°C. The nitrogen type/concentration was found to also have an impact on the molecular weights and polydispersity indices of the produced biopolymers. Furthermore, the tensile strengths were found to vary with the best tensile strength (14.4 MPa) being recorded for the polymer recovered from YE at C/N = 9. Interestingly, the tensile strength of PHBHV was significantly higher than petroleum‐based polyethylene (13.5 MPa), making it a much more suitable bioplastic for industrial application.

## Graphical abstract

This study aimed at investigating the impact of using inorganic (NH4Cl) and organic (yeast extract) as nitrogen sources on *Haloferax mediterranei* biomass formation, polyhydroxyalkanoate (PHA) accumulation, PHA composition on the monomeric level, PHA molecular mass, and PHA properties (thermal and mechanical).

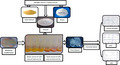

## 1 INTRODUCTION

At present, there is much attention being given to the disastrous pollution of the oceans, the origin of all life, by more than ten million tonnes of toxic petroleum‐derived plastics accumulated every year (Lebreton et al., 2017). Bioplastics are widely touted as a sustainable solution for reducing the environmental burden by partially replacing petroleum‐derived plastics. Bioplastic‐based polyhydroxyalkanoates (PHAs) are a family of polyesters that intracellularly accumulate as carbon and energy sources by several bacteria and archaea under unfavorable conditions (excess carbon and depletion of essential nutrients, such as nitrogen and oxygen) (Obruca, Sedlacek, Koller, Kucera, & Pernicova, 2018). PHAs have similar physical and mechanical properties to petroleum‐based plastics such as polypropylene (PP) and polyethylene (PE). A comprehensive life cycle assessment (LCA) study showed that PHAs are superior to petroleum‐derived plastics (PP and PE) in all major LCA categories if: (a) All process steps in the PHA life cycle are considered and optimized; (b) the industrial and ecological by‐products and wastes, and clean energy are used. (Narodoslawsky, Shazad, Kollmann, & Schnitzer, 2015). Moreover, PHAs are completely compostable and biodegradable in marine environments as certified by the standards of the American Society for Testing and Materials (ASTM) (Chen et al., 2017). It is noted that the biodegradation of PHA occurs either under aerobic conditions to produce carbon dioxide and water or under anaerobic conditions to produce methane and water (Altaee, El‐Hiti, Fahdil, Sudesh, & Yousif, 2016).

As a result of their ability to thrive in a saturated saline environment (∼4.5 M salt) with a water activity (a_w_) of 0.75, halophiles are attractive sources of PHAs that possess industrial applications (Alsafadi, Khalili, Juwhari, & Lahlouh, 2018; Grant, 2004; Koller, 2019; Van‐Thuoc et al., 2012). Compared with other PHA‐producing organisms, halophiles have unique advantages. Firstly, the high salinity of the halophilic microorganism's cultivation media minimizes the risk of microbial contamination (a_w_ of 0.6 is recognized as the lower limit for life) (Grant, 2004). Therefore, the cultivation environment can be simplified even without an expensive sterilization process. Secondly, the obtained PHA polymer can be easily recovered by a hypo‐osmotic shock of halophilic cells after decreasing the salinity of the external medium. Thirdly, the considerable amount of salts that result from the neutralization of the acid‐catalyzed hydrolysis process of raw materials (e.g., whey, rice straw, spent coffee ground, and liquefied wood) can contribute to the salinity of the fermentation medium (Koller, 2017).

Several halophilic microorganisms isolated from marine‐related niches can accumulate PHAs, albeit only a few have reached yields and volumetric productivities high enough to be considered practical for industrial purposes (Quillaguamán, Guzmán, Van‐Thuoc, & Hatti‐Kaul, 2010). Therefore, the biotechnological potential of halophiles for PHA production remains a topic for further inquiry. Thus far, the primary investigations reporting the production of PHAs from halophiles have used the extreme halophilic archaeon *Haloferax mediterranei* (*H. mediterranei*) as a model organism for the accumulation of PHAs from cheap carbon sources such as glycerol (Hermann‐Krauss et al., 2013), extruded starch (Chen, Don, & Yen, 2006), whey sugars (Koller et al., 2007), and olive mill wastewater (Alsafadi & Almashqbah, 2016).

Recent progress in the improvements of PHA biosynthesis from *H. mediterranei* has focused on optimizing the salt concentration of the medium (Cui, Gong, Shi, & Wang, 2018), studying the temperature of cultivation process (Cui, Shi, & Gong, 2017; Cui, Zhang, Ji, & Wang, 2017) and construction of mutant *H. mediterranei* strains by knocking out gene clusters that were involved in the synthesis of undesirable extracellular polymeric substances (Zhao et al., 2013). Attempts have also been made to produce effective amounts of PHA by controlling the substrate content (carbon and nitrogen ratio; C/N ratio). For example, the accumulation of PHA in *H. mediterranei* was optimal (~9.3% PHA/cell dry mass) at a C/N ratio of 8 with glucose and ammonium nitrate as a carbon and nitrogen source, respectively (Ferre‐Guell & Winterburn, 2017). *H. mediterranei* grows in the presence of different inorganic and organic nitrogen sources using the assimilatory pathway under aerobic conditions (Esclapez et al., 2015). Hence, to properly improve the biosynthesis of PHAs by *H. mediterranei*, we endeavored to further examine the influence of nitrogen from different sources on PHA accumulation and composition (monomer content). The *H. mediterranei* cultivation medium was supplemented by an inorganic N‐source (ammonium salt), or organic N‐source (yeast extract) with different C/N ratios (9, 20, or 35). The influence of nitrogen nutrients on the molecular weight of the produced polymers and their thermal and mechanical properties is also reported.

## 2 MATERIALS AND METHODS

### 2.1 Chemical reagent and standards

All chemical reagents, unless stated otherwise, were purchased as an analytical grade. Poly(3‐hydroxybutyric acid‐*co*‐3‐hydroxyvaleric acid) of natural origin (8 mol% 3HV), methyl (R)‐3‐hydroxybutyrate (99%), and methyl (R)‐3‐hydroxyvalerate (≥98.0%) were purchased from Sigma‐Aldrich. Monodisperse polystyrene standards (PStQuick C) for size exclusion analysis were purchased from TOSOH Corporation. Yeast extract and glucose were purchased from Oxoid.

### 2.2 Microorganisms and growth conditions

*Haloferax mediterranei* DSM 1,411 was obtained from the German Collection of Microorganisms and Cell cultures (DSMZ). The microorganism was initially grown in a liquid media (Koller, Chiellini, & Braunegg, 2015) containing (per liter) 150 g NaCl, 13 g MgCl_2_∙6H_2_O, 4 g KCl, 0.69 g CaCl_2_∙2H_2_O, 63 mg NH_4_Fe(III) citrate, 20 g MgSO_4_∙7H_2_O, 0.25 g NaHCO_3_, 0.5 g KBr, 6.25 g YE, and 10 g glucose and 1.25 ml SL‐6 solution containing 100 mg ZnSO_4_∙7H_2_O, 300 mg H_3_BO_3_, 200 mg CoCl_2_∙6H_2_O, 6 mg CuSO_4_, 20 mg NiCl_2_, 30 mg Na_2_MoO_4_∙2H_2_O, and 25 mg MnCl_2_∙2H_2_O. The media was adjusted to pH = 7.2 without sterilization before use. The culture was stored at −80°C in vials containing the same media and supplemented with 20% (*v*/*v*) glycerol. For solid media, *H. mediterranei* was grown at 37°C on agar plates containing the same liquid media compositions and with 15 g/L agar.

### 2.3 PHA production

A single *H. mediterranei* colony grown on solid media was inoculated into 100 ml of the liquid media. The media was adjusted to pH = 7.2 and not sterilized before use. The culture was incubated with constant shaking (230 rpm) at 37°C. When the cells had reached the late exponential phase (monitored by the achievement of an optical density at 520 nm), 3 ml of a selected preculture was transferred into 100 ml of liquid modified medium. Three sets of liquid modified media, NH_4_Cl, YE, and a combination of YE and NH_4_Cl were prepared with differing initial nitrogen concentrations. To achieve the desired C/N ratio, the initial glucose concentration (10 g/L) was kept constant, while the amount of nitrogen was reduced by decreasing the amount of NH_4_Cl or YE. In the NH_4_Cl media, 1.70, 0.77, and 0.44 g/L of NH_4_Cl was added to achieve C/N ratios of 9, 20, and 35, respectively. Before formulating the YE media, the carbon and nitrogen content in yeast extract was determined by an elemental analyzer. The YE contained 11.4% total nitrogen and 39.9% carbon. Therefore, 6.5, 2.15 and 1.1 g/L of YE were added to achieve C/N ratios of 9, 20 and 35, respectively. In the media containing YE and NH_4_Cl, (0.85 g/L NH_4_Cl and 3.25 g/L YE), (0.39 g/L NH_4_Cl and 1.08 g/L YE) and (0.22 g/L NH_4_Cl and 0.55 g/L YE) were added to achieve C/N ratios of 9, 20, and 35, respectively. To estimate the direct effect of YE as a carbon and nitrogen source, *H. mediterranei* was cultivated in glucose‐free media containing the same liquid medium compositions and without glucose. The production of PHA was carried out in 250 ml Erlenmeyer flasks for 4 days with shaking at 230 rpm and 37°C.

### 2.4 PHA extraction and analysis

Polyhydroxyalkanoate extraction was performed as described previously (Alsafadi & Almashqbah, 2016). PHA content was quantified by gas chromatography (GC Shimadzu 2010 equipped with a flame ionization detector). A standard calibration curve was established using standard poly(3‐hydroxybutyric acid‐*co*‐3‐hydroxyvaleric acid) 8 mol% 3HV from Sigma‐Aldrich with benzoic acid serving as an internal standard (Alsafadi & Almashqbah, 2016). Retention times were 20.7 min for methylated 3‐hydroxybutyric acid, 24.7 min for methylated 3‐hydroxyvaleric acid, and 27.1 min for methylated benzoic acid.

### 2.5 Determination of cell growth and cell dry mass

The cell growth was analyzed spectrophotometrically by measuring the optical density at 520 nm (OD_520 nm_), using a Biochrom Libra S50 UV–visible spectrophotometer. The media was used as a blank for the OD measurements. To determine the cell dry mass (CDM), 3 ml of the broth containing *H. mediterranei* cells was centrifuged at 6,340 × *g* for 15 min and then the supernatant was discarded. The remaining pellet was washed twice with an isotonic NaCl solution. The pellet was dried in an oven at 105°C to reach a constant mass.

### 2.6 Analytical methods

Carbon and nitrogen content were determined by the elemental analyzer Eurovector Co. (model E.A 3000). Molar mass data for PHA were calculated from gel permeation chromatography (GPC) measurements on a Tosho HLC‐8320 GPC (EcoSEC) equipped with a refractive index detector and TSKgel GMH_HR_‐M column (7.8 mm I.D × 30 cm and 5 μm particle size). The pump and column ovens were set at 35°C, and chloroform was used as the eluent at a flow rate of 1.0 ml/min. Monodisperse polystyrene standards (PStQuick C) were used to prepare the calibration curve for a working range of 500 Da–2110 kDa. The PHA samples (12.0 mg) were dissolved in chloroform (2.0 ml), and the molar mass data (number average molecular weight (*M*_w_) and weight average molecular weight (*M*_n_) and polydispersity (*M*_w_/*M*_n_]) were calculated by the use of standard analysis software (EcoSEC Logon Manager Version 1.02). The extracellular polymeric substances (EPSs) were determined by taking the difference between total carbohydrate and glucose concentrations. The total carbohydrate was determined by the Anthrone‐Sulfuric Acid Method (Koller et al., 2015). The glucose concentration was determined using a Thermo UltiMate 3000 HPLC equipped with a refractive index detector and ACE‐Excel column. Glucose monohydrate standards of defined concentrations were used for external calibration.

### 2.7 Mechanical and thermal properties analysis

For the tensile strength measurement, the purified PHA samples were dissolved in chloroform and heated to boiling in a covered beaker under constant stirring until the samples were completely dissolved. A polymer solution with a concentration of 0.02 g/ml of PHA in chloroform was used to prepare all test films. Approximately, 10 ml of the polymer solution was poured on a glass slide (70 mm × 35 mm). The solution was dried at 25°C for 24 hr to prepare film samples. The PHA films were cut into regular shape with a width of 4 mm. The thickness of the film samples was measured using a digital thickness gauge (Rainbow Karl Schroeder KG). PHA films with an average of 0.1 mm thickness were obtained. The tensile strengths were measured at a crosshead speed of 0.2 mm/min using a Shimadzu tensile strength machine (Model AG‐IS 5kN). The thermal properties of the PHA were examined by differential scanning calorimetry using a Netzsch DSC 200 F3 Maia. PHA samples were exposed to a temperature profile over 0–220°C at a heating rate of 10°C/min. Nitrogen gas was purged into the sample with a flow rate of 20 ml/min.

## 3 RESULTS AND DISCUSSION

### 3.1 Effect of nitrogen on *H. mediterranei* cell growth

**FIGURE 1:**
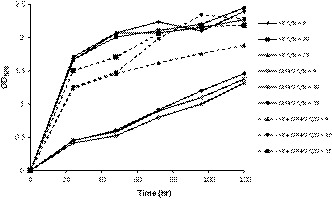
Growth time profile *of Haloferax mediterranei* at different nitrogen source and C/N ratio

**FIGURE A1:**
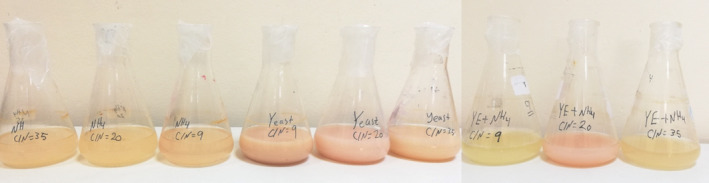
Final culture broth of *Haloferax mediterranei* with different nitrogen sources and C/N ratios

In this study, different *H. mediterranei* liquid media were prepared using glucose as the sole carbon source along with two different types of nutrients serving as the nitrogen sources (NH_4_Cl and YE). It is noted that the use of YE as a nitrogen source has the potential to introduce variability as its composition is unknown, therefore, carbon and nitrogen contents for YE were determined by the elemental analyzer, and the results were used in the formulation of the medium at different C/N ratios of 9, 20, and 35 as described in the material and method part. Figure 1 shows the growth profile of *H. mediterranei* in liquid media containing different nitrogen sources at different C/N ratios (9, 20, and 35). The growth profile in media containing YE (YE and combined media) produced the expected exponential normal growth (log phase) of *H. mediterranei* whereas the growth profile in NH_4_Cl media exhibited a linear line with the very slow growth of the organism after 120 hr. The results clearly show that the growth of *H. mediterranei* was more impacted by the source of nitrogen than to the availability of the nitrogen. The media containing YE (organic nitrogen source) produced a higher growth rate of *H. mediterranei* than NH_4_Cl (inorganic source) at all tested C/N ratios (9, 20, and 35). The optimal cell growth was recorded at C/N ratios of 9 and 20 in media containing YE. Interestingly, the growth rate of *H. mediterranei* in the media formulated from the combination of YE and NH_4_Cl was dependent on the C/N ratio. A high growth rate was recorded for C/N ratios = 20 and 35 while *H. mediterranei* showed slower growth at a C/N ratio = 9. These results are corroborated by the image of the final cultures broth of *H. mediterranei* with different nitrogen sources and C/N ratios (Appendix Figure A1). A highly‐dense pinkish color with mucous characteristics for *H. mediterranei* growth was observed from YE media at C/N ratios of 9 and 20. The pinkish color with mucous characteristics was also observed for *H. mediterranei* after 48 hr of cultivation on solid media containing yeast extract plus a defined carbon source (carbohydrates) (Koller et al., 2015). This could be attributed to the formation of the extracellular polymeric substances in parallel with PHA during the *H. mediterranei* cultivation.

### 3.2 Impact of nitrogen on PHA production and 3‐hydroxyvalerate mole ratio

Following PHA production, *H. mediterranei* cells were harvested and PHA was extracted and purified as described in the method part. The data for PHA concentration for different nitrogen sources (YE and NH_4_Cl) and different C/N ratios (9, 20, 35) are presented in Table 1. The cell dry mass (CDM) and PHA concentration of *H. mediterranei* cultivated in media containing YE were higher than NH_4_Cl media. The high PHA production values are because YE used as a complex organic source of nitrogen (amino acids and peptides), was metabolized into biomass. This reduces the energy cost of the cell to synthesize amino acids and, consequently, active cell mass is built up rapidly with a short lag phase, thereby, increasing PHA production. Nevertheless, the effect of YE as a nitrogen source on PHA production by *H. mediterranei* has not been reported yet in the literature, although it might be important in revealing pathways for increasing PHA production efficiency. Previous studies have investigated the effect of nitrogen on *H. mediterranei* cell growth and PHA production using inorganic nitrogen sources such as ammonium and nitrate salts (Cui, Shi, & Gong, 2017; Cui, Zhang, Ji, & Wang, 2017; Ferre‐Guell & Winterburn, 2017). Ferre‐Guell and Winterburn (2017) reported that the cultivation of *H. mediterranei* in ammonium and nitrate salt‐based media with excess nitrogen (C/N = 8) generated 10.7 g/L biomass containing 4.6% PHA and 5.6 g/L biomass containing 9.3% PHA, respectively. Here, *H. mediterranei* cultivated in YE medium at C/N = 9 showed a higher CDM (~13 g/L) and polymer accumulation (14.4%) than the values reported for *H. mediterranei* grown in inorganic nitrogen sources under similar conditions (i.e., batch flask fermentations). YE could be used as a nitrogen source and carbon source as well to support *H. mediterranei* and PHA production. To estimate the direct effect of YE as a carbon and nitrogen source, *H. mediterranei* was cultivated in glucose‐free media (materials and methods). Under this condition, 0.57 g/L PHA was obtained, which was significantly higher than the results when using the NH_4_Cl media (Table 1). This result confirmed that YE was a more suitable nitrogen source for better *H. mediterranei* cell growth and PHA production than the inorganic ammonium nitrogen source. YE is widely used as a growth stimulant or growth factor for bacteria and archaea. Moreover, YE has been evaluated as the best nitrogen source for PHA production and cell growth for *Bacillus sp*. CFR 67 (Sreekanth, Vijayendra, Joshi, & Shamala, 2012) and *Comamonas sp*. EB172 (Zakaria, Ariffin, Abd‐Aziz, Hassan, & Shirai, 2013). In further experiments, *H. mediterranei* was cultivated in media formulated form the combination of YE and NH_4_Cl (Table 1). In this experiment, the obtained CDM and PHA were significantly higher than the results of the NH_4_Cl media (Table 1). More importantly, the results obtained from the cultivation of *H. mediterranei* in the combined media show that the use of NH_4_Cl as a simple nitrogen source and YE as a complex nitrogen source could improve PHA production. This observation was very clear at a C/N ratio = 20, where the highest polymer accumulation (18.4%) was obtained as shown in Table 1. It is noted that the price of YE is much higher than inorganic nitrogen sources such as ammonium and nitrate salts. The results of the combined media are very promising where the amount of expensive YE could be reduced without affecting the production of PHA by *H. mediterranei*. As expected, the obtained CDM from YE media increased with increasing nitrogen concentration while the PHA concentration did not display a similar trend (Table 1). The N‐deficient conditions at C/N ratios of 35 inhibited *H. mediterranei* cell growth and decreased the PHA production to 1.45 g/L. When the PHA concentrations at C/N ratios of 20 and 9 were compared, the PHA concentration decreased from 2.12 to 1.90 g/L with decreasing C/N ratios. It has been reported that *H. mediterranei* also produce extracellular polymeric substances (EPSs) simultaneously with PHA synthesis (Cui, Shi, & Gong, 2017; Cui, Zhang, Ji, & Wang, 2017; Koller et al., 2015). These studies have suggested that EPSs synthesis may reduce PHA production. Clearly, in YE media the process for EPSs synthesis was induced under surplus nitrogen condition with a C/N ratio of 9, thereby, decreasing PHA production to 1.9 g/L. This result was confirmed by EPSs concentration in the supernatant of the YE media (1.0 g/L at C/N = 9 and 0.4 g/L at C/N = 20). In NH_4_Cl media, the PHA accumulation generally increased with increasing C/N ratio at fixed glucose concentration. A similar effect was also observed with *Cupriavidus necator* DSM 545. For this organism, the PHA productivity increased from 0.15 to 0.72 g/L with increasing C/N ratios from 3.6 to 360 (Ahn, Jho, & Nam, 2015). In YE and NH_4_Cl combined cultures, *H. mediterranei* showed reduced biomass and polymer concentration under excess nitrogen (C/N = 9) and nitrogen‐limiting (C/N = 35) conditions. However, the CDM and polymer concentration increased to 12.5 g/L and 2.30 g/L at a C/N ratio = 20, respectively.

**TABLE 1:** Effect of nitrogen on PHA production and 3‐hydroxyvalerate mol ratio after 96 hr of cultivation

Nitrogen Source	C/N	CDM (g/L)	PHA (g/L)	Residual biomass (g/L)	% PHA (g/g)	3HV mol%
YE	9	12.85 ± 0.4	1.90 ± 0.06	10.95	14.4 ± 0.4	9.1
YE	20	12.10 ± 0.3	2.12 ± 0.05	9.98	17.7 ± 0.4	10.2
YE	35	10.5 ± 0.4	1.45 ± 0.07	9.05	13.8 ± 0.5	6.2
NH_4_Cl	9	4.75 ± 0.3	0.19 ± 0.05	4.56	4.0 ± 0.3	1.2
NH_4_Cl	20	4.9 ± 0.2	0.27 ± 0.08	4.63	5.5 ± 0.3	0.6
NH_4_Cl	35	5.7 ± 0.2	0.40 ± 0.08	5.3	7.0 ± 0.4	1.4
YE + NH_4_Cl	9	10.30 ± 0.3	1.01 ± 0.01	9.29	9.8 ± 0.4	9.5
YE + NH_4_Cl	20	12.50 ± 0.3	2.3 ± 0.02	10.2	18.4 ± 0.3	10
YE + NH_4_Cl	35	9.25 ± 0.4	1.48 ± 0.01	7.77	16.0 ± 0.4	9.5

Note

The results were presented in mean ± *SD*.

*Haloferax mediterranei* has the capability of biosynthesis the copolymer poly(3‐hydroxybutyrate‐*co*‐3‐hydroxyvalerate) (PHBHV) from cheap carbon sources with a chemical structure unrelated to 3‐hydroxyvalerate (3HV), such as glycerol (Hermann‐Krauss et al., 2013) and carbohydrates (Han et al., 2013). This biosynthesis was elucidated by Han et al. (2013) who demonstrated that *H. mediterranei* can resort to multiple supplying pathways in the synthesis of propionyl coenzyme A (important precursor of 3HV monomer). This includes the citramalate/2‐oxobutyrate, aspartate/2‐oxobutyrate, methylmalonyl‐CoA, and 3‐hydroxypropionate pathways. The ratio of 3HV in PHBHV is important for increasing properties that are relevant to the industrial application of the copolymer (*e*.*g*., flexibility and impact strength). The results presented in Table 1 indicate that the nitrogen source plays a key role in controlling 3HV content in PHBHV copolymer produced by *H. mediterranei*. The produced polymers from YE and combined media have 3HV mol% greater than polymer recovered from NH_4_Cl media (Table 1). In both media, the highest 3HV mol% was reported at C/N = 20 with ~10 mol% of 3HV. This 3HV content was considerably similar to values reported for PHBHV produced by *H. mediterranei* (Chen, Don, & Yen, 2006; Hermann‐Krauss et al., 2013; Koller, 2015; Koller et al., 2015). The effect of 3HV content on the thermal and mechanical properties of the copolymer will be investigated in the next part of this study.

### 3.3 Impact of nitrogen on molecular weight, thermal and mechanical properties of PHA

The molecular weight data for PHA was determined using size exclusion chromatography as described previously in the method part. Table 2 shows that the molecular weights (*M*_w_) and polydispersity indices (PDI; *M*_w_/*M*_n_) of PHA also varied with the types of nitrogen and C/N ratios (9, 20 and 35) used in the fermentation. While C/N = 20 would be one of the best ratios for PHA production in YE media, a low value of *M*_w_ (276 kDa) was observed for the C/N ratio of 20. The highest *M*w for PHBHV was recorded at 1,014 kDa with a PDI value of 1.8 using yeast extract at a C/N ratio = 9. This *Mw* is in a similar to measured molecular mass for the high‐quality PHBHV produced by *H. mediterranei (Mw* = 1,057 kDa) (Koller et al., 2007). Further, the PDI was close to the PDI value of PHBV biosynthesized by *H. mediterranei* which was reported by (Koller et al., 2007) (PDI = 1.50) and (Han, Wu, Hou, Zhao, & Xiang, 2015) (PDI = 1.63). Interestingly, the PDI (1.8) of the polymer recovered from YE at C/N = 9 is much lower than that of polymer recovered from C/N = 20 (PDI = 5.8) and C/N = 35 (PDI = 4.9), respectively. This is suggesting that the polymer recovered at C/N = 9 is more homogeneous in the chain length. In NH_4_Cl media, the highest *M*_w_ (908 KDa) was recorded at the C/N ratio of 20 with narrow molecular weight distribution (PDI = 2.0). For those cultures formulated from the combination of YE and NH_4_Cl, the highest M_w_ (211 KDa) was recorded at a C/N ratio = 20 with molecular weight distribution (PDI 3.5).

**TABLE 2:** Effect of nitrogen on molecular weight, thermal and mechanical properties of polymer produced by *Haloferax mediterranei* after 96 hr of cultivation

Nitrogen Source	C/N	Molecular weight^a^		Thermal properties (°C)^b^			Mechanical properties^c^
		*M*_w_ (kDa)	PDI [*M*_w_/*M*n]	*T*_g_	*T*_m1_	*T*_m2_	Tensile (MPa)
YE	9	1,014	1.8	1.2	147.5	ND	14.4
YE	20	276	5.8	ND	140	148.1	2.8
YE	35	277	4.9	1.5	144.6	157.2	1.1
NH_4_Cl	9	493	2.2	−7.5	156.2	ND	7.3
NH_4_Cl	20	908	2	−2.7	151.9	ND	3.7
NH_4_Cl	35	759	1.8	−6.1	158.2	ND	1.9
YE + NH_4_Cl	9	134	3.3	ND	136.9	1525	ND
YE + NH_4_Cl	20	211	3.5	ND	139.4	153.9	2.4
YE + NH_4_Cl	35	70	2.9	ND	136.6	154.1	ND

^a^ *M*_w_, weight average molecular weight; PDI, polydispersity index [*M*_w_/*M*_n_].

^b^ *T*_g_, glass transition temperature; *T*_m_, melting temperature; ND, not detectable.

^c^ Tensile strength.

From previous studies, it was reported that the molecular weight of PHA depends on several factors. For example, Sim et al. (1997) reported that the molecular weight and the PDI of PHA depend on the activity of PHA synthase (the key enzyme in PHA biosynthesis). On the other hand, Dennis, McCoy, Stangl, Valentin, and Wu (1998) claims that the molecular weight would not be a sole function of PHA synthase's activity, but, rather, of the relation between PHA synthase's activity and substrate availability. Quagliano, Amarilla, Fernandes, Mata, and Miyazaki (2001) reported that the carbon source (complex or simple) was solely responsible for the PHA molecular weight. For example, the molar masses of PHA produced by *R. eutropha* (Madden, Anderson, Shah, & Asrar, 1999) and *H. mediterranei* (Hermann‐Krauss et al., 2013) from glycerol as sole carbon source were substantially lower than biopolymer produced from glucose. The reduction in molar masses was attributed to glycerol molecule which bound to the PHA chain (via covalent linking at the carboxyl terminus of PHA) and causes a termination of chain propagation “end‐capping effect” (Hermann‐Krauss et al., 2013). Here, this study shows that different types and/or concentrations of nitrogen sources in the cultivation media also have a direct effect on controlling the molecular weight and molecular weight distribution of PHA. The ability to control the molecular weight of PHA is an advantage in polymer manufacturing and processing as well as it offers a wide range of industrial applications for PHA in the plastic industry.

DSC analysis in Table 2 demonstrates the measured melting points for the polymers recovered from YE, and the combined media were lower than the melting point of polymers from NH_4_Cl media. This result was consistent with the 3HV monomer composition of the copolymer PHBHV (Table 1) as a larger amount of 3HV incorporated into PHBHV could lead to a lower *T*_m_. The low melting point of the copolymers could improve its processing ability and impact its strength. Additionally, the thermal analysis revealed a glass transition point (*T*_g_) range from (−6.1 to 1.5°C) (Table 2). These data are very similar to reported *T*_g_ values for PHBHV produced by *H. mediterranei* (Han et al., 2010; Koller et al., 2007).

In a further experiment, the tensile strength of the polymers was investigated at room temperature (Table 2). The polymer recovered from YE with a C/N ratio of 9 recorded the best tensile strength (14.4 MPa). This tensile strength is higher than the tensile strength of petroleum‐based PE (13.5 MPa) (Liu, Zhang, Dego, & Zhao, 2014). It has been reported that the main influence on the tensile strength of PHBHV is the content of the 3HV monomer unit in the copolymer (Liu et al., 2014). Although the increase of the 3HV mol% in the PHBHV increases the flexibility and the impact strength, a sharp reduction in the tensile strength was observed in a practical application of the copolymer (Conti, Pezzin, & Coelho, 2007). According to this hypothesis, YE‐derived polymers with high 3HV mol% (6–10) are expected to have low tensile strength; however, it seems that the *M*_w_ and PDI are the main limiting factors in improving the tensile strength for the YE‐derived polymers (Table 1). Many efforts have been performed to improve the mechanical properties of PHBHV including, blending PHBHV with polymers, natural fibers, carbon nanomaterials, nitrocellulose, nanoclays, and nanometals (Rivera‐Briso & Serrano‐Aroca, 2018). In this study, we have reported that the optimization of the nitrogen source in different cultivation media of *H. mediterranei* has the potential to enhance the mechanical properties of the produced PHBHV.

## 4 CONCLUSION

*Haloferax mediterranei* an extremely halophilic archaeon has been reported to have the ability for accumulating copolymer PHBHV from unrelated and simple carbon sources. The results of this study revealed that the production, composition on the monomeric level, and properties (thermal and mechanical) of PHBHV can be enhanced by controlling the nitrogen source (YE and NH_4_Cl) and C/N ratio (9, 20, 35). The study also demonstrated for the first time the feasibility of combining YE and NH_4_Cl as nitrogen sources for PHA production using *H. mediterranei*. Implementation of this strategy resulted in an increase in the CDM and PHA concentration to 12.5 g/L and 2.30 g/L, respectively, at a C/N ratio = 20. The produced PHBHV copolymers from YE media displayed reduced melting points (~144°C), which potentially result in improvement in the impact strength and flexibility of the copolymer. This is mainly due to the high 3HV content (6–10 mol%) in copolymer chains. The molecular weights (*M*_w_) and polydispersity index (PDI; *M*_w_/*M*_n_) of the polymers significantly varied for different types and/or concentrations of nitrogen sources. The highest *M*_w_ for PHBHV was reached at 1,014 kDa with a PDI value of 1.8 using yeast extract at a C/N ratio = 9. This value of *M*_w_ is very promising for making PHBHV produced by *H. mediterranei* potential candidate for industrial applications. Studying the mechanical properties of the produced polymers is important to provide information about the characteristics of the material before its use in industrial settings. Interestingly, PHBHV produced from YE media with a C/N ratio = 9 exhibited higher tensile strength than the petroleum‐based PE. Overall, the quantity and properties of the PHAs produced by *H. mediterranei* have been improved using YE as a nitrogen source. The price of YE (market value approximately 50 € kg^−1^) is much higher than inorganic nitrogen sources such as ammonium and nitrate salts. However, the high PHBHV production with excellent polymer characteristics (low melting temperature, high tensile strength and high molecular masses with narrow distribution) makes the YE a reasonable choice for use as a nitrogen source for PHBHV production by *H. mediterranei*. For realizing the industrial‐scale production of PHBHV using *H. mediterranei,* future work should focus on (a) scale‐up the process using continuous feeding of the nutrients under the optimized conditions (nitrogen source and C/N); (b) engineering *H. mediterranei* to enlarge cell sizes for more PHBHV accumulation space; (c) reuse the remaining saline *H. mediterranei* fermentation media after optimizing its C/N ratio.

## CONFLICT OF INTERESTS

None declared.

## AUTHOR CONTRIBUTION

**Diya Alsafadi:** Conceptualization (lead); Funding acquisition (lead); Methodology (equal); Validation (equal); Writing‐original draft (lead). **Othman Al‐Mashaqbeh:** Data curation (supporting); Funding acquisition (supporting); Investigation (equal); Methodology (equal); Supervision (supporting); Writing‐original draft (supporting). **Aya Mansour:** Data curation (supporting); Formal analysis (supporting); Investigation (supporting); Methodology (supporting); Resources (supporting). **Majd Alsaad:** Data curation (supporting); Investigation (supporting); Methodology (supporting).

## ETHICS STATEMENT

None required.

## Data Availability

All data generated or analyzed during this study are included in this published article.

## References

[mbo31055-bib-0001] Ahn, J. , Jho, E. H. , & Nam, K. (2015). Effect of C/N ratio on polyhydroxyalkanoates (PHA) accumulation by cupriavidus necator and its implication on the use of rice straw424 hydrolysate. Environmental Engineering Research, 20, 246–253.

[mbo31055-bib-0002] Alsafadi, D. , & Almashqbah, O. (2016). A one‐stage cultivation process for the production of poly‐3‐(hydroxybutyrate‐co‐hydroxyvalerate) from olive mill wastewater by *Haloferax mediterranei* . New Biotechnology, 43, 47–53. 10.1016/j.nbt.2016.05.003 27224675

[mbo31055-bib-0003] Alsafadi, D. , Khalili, F. , Juwhari, H. , & Lahlouh, B. (2018). Purification and biochemical characterization of photo‐active membrane protein bacteriorhodopsin from haloarcula marismortui, an extreme halophile from the Dead sea. International Journal of Biological Macromolecules, 118, 1942–1947. 10.1016/j.ijbiomac.2018.07.045 30017983

[mbo31055-bib-0004] Altaee, N. , El‐Hiti, G. A. , Fahdil, A. , Sudesh, K. , & Yousif, E. (2016). Biodegradation of different formulations of polyhydroxybutyrate films in soil. Springerplus, 762, 1–12. 10.1186/s40064-016-2480-2 PMC4912537 27386248

[mbo31055-bib-0005] Chen, C. W. , Don, T. M. , & Yen, H. F. (2006). Enzymatic extruded starch as a carbon source for the production of poly (3‐hydroxybutyrate‐co‐3‐hydroxyvalerate) by *Haloferax mediterranei* . Process Biochemistry, 41, 2289–2296. 10.1016/j.procbio.2006.05.026

[mbo31055-bib-0006] Chen, X. , Yin, J. , Ye, J. , Zhang, H. , Che, X. , Ma, Y. , … Chen, G. Q. (2017). Engineering *Halomonas bluephagenesis* TD01 for non‐sterile production of poly(3‐hydroxybutyrate‐co‐4‐hydroxybutyrate). Bioresource Technology, 244, 534–541. 10.1016/j.biortech.2017.07.149 28803103

[mbo31055-bib-0007] Conti, D. S. , Pezzin, S. H. , & Coelho, L. A. F. (2007). Mechanical and morphological properties of Poly(3‐hydroxybutyrate)/ Poly(3‐hydroxybutyrate‐co‐3‐hydroxyvalerate) blends. Macromolecular Symposium, 245–246, 491–500.

[mbo31055-bib-0008] Cui, Y. W. , Gong, X. Y. , Shi, Y. P. , & Wang, Z. (2018). Salinity effect on production of PHA and EPS by *Haloferax mediterranei* . RSC Advances, 7, 53587–53595.

[mbo31055-bib-0009] Cui, Y.‐W. , Shi, Y.‐P. , & Gong, X.‐Y. (2017). Effects of C/N in the substrate on the simultaneous production of polyhydroxyalkanoates and extracellular polymeric substances by *Haloferax mediterranei* via kinetic model analysis. RSC Advances, 7, 18953–18961. 10.1039/C7RA02131C

[mbo31055-bib-0010] Cui, Y.‐W. , Zhang, H.‐Y. , Ji, S.‐Y. , & Wang, Z.‐W. (2017). Kinetic analysis of the temperature effect on polyhydroxyalkanoate production by *Haloferax mediterranei* in synthetic molasses wastewater. Journal of Polymers and the Environment, 25, 277–285. 10.1007/s10924-016-0807-2

[mbo31055-bib-0011] Dennis, D. , McCoy, M. , Stangl, A. , Valentin, H. E. , & Wu, Z. (1998). Formation of poly(3‐hydroxybutyrate‐co‐3‐hydroxyhexanoate) by PHA synthase from ralstonia eutropha. Journal of Biotechnology, 64, 177–186. 10.1016/S0168-1656(98)00110-2 9821674

[mbo31055-bib-0012] Esclapez, J. , Pire, C. , Camacho, M. , Bautista, V. , Martínez‐Espinosa, R. M. , Zafrilla, B. , … Bonete, M. J. (2015). Transcriptional profiles of *Haloferax mediterranei* based on nitrogen availability. Journal of Biotechnology, 193, 100–107. 10.1016/j.jbiotec.2014.11.018 25435380

[mbo31055-bib-0013] Ferre‐Guell, A. , & Winterburn, J. (2017). Production of the copolymer poly(3‐hydroxybutyrate‐co‐3‐hydroxyvalerate) with varied composition using different nitrogen sources with *Haloferax mediterranei* . Extremophiles, 6, 1037–1047. 10.1007/s00792-017-0964-9 28988336

[mbo31055-bib-0014] Grant, W. D. (2004). Life at low water activity. Philosophical Transactions of the Royal Society of London. Series B, Biological Sciences, 359, 1249–1267. 10.1098/rstb.2004.1502 15306380 PMC1693405

[mbo31055-bib-0015] Han, J. , Hou, J. , Zhang, F. , Ai, G. , Li, M. , Cai, S. , … Xiang, H. (2013). Multiple propionyl coenzyme A‐supplying pathways for production of the bioplastic poly(3‐hydroxybutyrate‐co‐3‐hydroxyvalerate) in *Haloferax mediterranei* . Applied and Environmental Microbiology, 79, 2922–2931. 23435886 10.1128/AEM.03915-12 PMC3623125

[mbo31055-bib-0016] Han, J. , Li, M. , Hou, J. , Wu, L. , Zhou, J. , & Xiang, H. (2010). Comparison of four phaC genes from *Haloferax mediterranei* and their function in different PHBHV copolymer biosyntheses in *Haloarcula hispanica* . Saline Systems, 6, 1–9. 20727166 10.1186/1746-1448-6-9 PMC2939530

[mbo31055-bib-0017] Han, J. , Wu, L. P. , Hou, J. , Zhao, D. , & Xiang, H. (2015). Biosynthesis, characterization and hemostasis potential of tailor‐made Poly (3‐hydroxybutyrate‐co‐3‐hydroxyvalerate) produced by Haloferax mediterranei. Biomacromolecules, 16, 578–588. 10.3390/bioengineering2020094. 25559462

[mbo31055-bib-0018] Hermann‐Krauss, C. , Koller, M. , Muhr, A. , Fasl, H. , Stelzer, F. , & Braunegg, G. (2013). Archaeal production of polyhydroxyalkanoate (PHA) co‐ and terpolyesters from biodiesel industry derived by‐products. Archaea, 2013, 1–10. 10.1155/2013/129268 PMC3880725 24453697

[mbo31055-bib-0019] Koller, M. (2015). Recycling of waste streams of the biotechnological poly(hydroxyalkanoate) production by *Haloferax mediterranei* on whey. International Journal of Polymer Science, 2015, 1–8.

[mbo31055-bib-0020] Koller, M. (2017). Production of polyhydroxyalkanoate (PHA) biopolyesters by extremophiles. MOJ Polymer Science, 1, 1–19. 10.15406/mojps.2017.01.00011

[mbo31055-bib-0021] Koller, M. (2019). Polyhydroxyalkanoate biosynthesis at the edge of water activitiy‐haloarchaea as biopolyester factories. Bioengineering, 6, 1–33. 10.3390/bioengineering6020034 PMC6631277 30995811

[mbo31055-bib-0022] Koller, M. , Chiellini, E. , & Braunegg, G. (2015). Study on the production and re‐use of Poly(3‐hydroxybutyrate‐co‐3‐hydroxyvalerate) and extracellular polysaccharide by the archaeon *Haloferax mediterranei* Strain DSM 1411. Chemical and Biochemical Engineering Quarterly, 29, 87–98. 10.15255/CABEQ.2014.2058

[mbo31055-bib-0023] Koller, M. , Hesse, P. , Bona, R. , Kutschera, C. , Atlić, A. , & Braunegg, G. (2007). Biosynthesis of high quality polyhydroxyalkanoate co‐ and terpolyesters for potential medical application by the archaeon *Haloferax mediterranei* . Macromolecular Symposium, 253, 33–39. 10.1002/masy.200750704

[mbo31055-bib-0024] Lebreton, L. C. M. , van der Zwet, J. , Damsteeg, J.‐W. , Slat, B. , Andrady, A. , & Reisser, J. (2017). River plastic emissions to the world's oceans. Nature Communications, 8, 15611–15620. 10.1038/ncomms15611 PMC5467230 28589961

[mbo31055-bib-0025] Liu, Q. , Zhang, H. , Dego, B. , & Zhao, X. (2014). Poly(3‐hydroxybutyrate) and poly(3‐hydroxybutyrate‐co‐3‐hydroxyvalerate): Structure, property, and fiber. International Journal of Polymer Science, 2014, 1–11. 10.1155/2014/374368

[mbo31055-bib-0026] Madden, L. A. , Anderson, A. J. , Shah, D. T. , & Asrar, J. (1999). Chain termination in polyhydroxyalkanoate synthesis: Involvement of exogenous hydroxy‐compounds as chain transfer agents. International Journal of Biological Macromolecules, 25, 43–53. 10.1016/S0141-8130(99)00014-8 10416649

[mbo31055-bib-0027] Narodoslawsky, M. , Shazad, K. , Kollmann, R. , & Schnitzer, H. (2015). LCA of PHA production identifying the ecological potential of bio‐plastic. Chemical and Biochemical Engineering Quarterly, 29, 299–305. 10.15255/CABEQ.2014.2262

[mbo31055-bib-0028] Obruca, S. , Sedlacek, P. , Koller, M. , Kucera, D. , & Pernicova, I. (2018). Involvement of polyhydroxyalkanoates in stress resistance of microbial cells: Biotechnological consequences and applications. Biotechnology Advances, 36, 856–870. 10.1016/j.biotechadv.2017.12.006 29248684

[mbo31055-bib-0029] Quagliano, J. C. , Amarilla, F. , Fernandes, E. G. , Mata, D. , & Miyazaki, S. S. (2001). Effect ofsimple and complex carbon sources, low temperature culture and complex carbon feedingpolicies on poly‐3‐hydroxybutyric acid (PHB) content and molecular weight from azotobacter chroococcum 6B. World Journal of Microbiology and Biotechnology, 17, 9–14.

[mbo31055-bib-0030] Quillaguamán, J. , Guzmán, H. , Van‐Thuoc, D. , & Hatti‐Kaul, R. (2010). Synthesis and production of polyhydroxyalkanoates by halophiles: Current potential and future prospects. Applied Microbiology and Biotechnology, 85, 1687–1696. 10.1007/s00253-009-2397-6 20024541

[mbo31055-bib-0031] Rivera‐Briso, A. L. , & Serrano‐Aroca, Á. (2018). Poly(3‐hydroxybutyrate‐co‐3 hydroxyvalerate): Enhancement strategies for advanced. Applications Polymers, 10, 1–28. 10.3390/polym10070732 PMC6403723 30960657

[mbo31055-bib-0032] Sim, S. J. , Snell, K. D. , Hogan, S. A. , Stubbe, J. A. , Rha, C. , & Sinskey, A. J. (1997). PHA synthase activity controls the molecular weight and polydispersity of polyhydroxybutyrate in vivo. Nature Biotechnology, 15, 63–67. 10.1038/nbt0197-63 9035108

[mbo31055-bib-0033] Sreekanth, M. S. , Vijayendra, S. V. , Joshi, G. J. , & Shamala, T. R. (2012). Effect of carbon and nitrogen sources on simultaneous production of α‐amylase and green food packaging polymer by *Bacillus* sp. CFR 67. Journal of Food Science and Technology, 50, 404–408. 24425935 10.1007/s13197-012-0639-6 PMC3550928

[mbo31055-bib-0034] Van‐Thuoc, D. , Huu‐Phong, T. , Thi‐Binh, N. , Thi‐Tho, N. , Minh‐Lam, D. , & Quillaguamán, J. (2012). Polyester production by halophilic and halotolerant bacterial strains obtained from mangrove soil samples located in Northern Vietnam. Microbiologyopen, 1, 395–406. 10.1002/mbo3.44 23233461 PMC3535385

[mbo31055-bib-0035] Zakaria, M. R. , Ariffin, H. , Abd‐Aziz, S. , Hassan, M. A. , & Shirai, Y. (2013). Improved properties of poly(3‐hydroxybutyrate‐co‐3‐hydroxyvalerate) produced by *Comamonas* sp. EB172 utilizing volatile fatty acids by regulating the nitrogen source. BioMed Research International, 2013, 1–7. 10.1155/2013/237806 PMC3784149 24106698

[mbo31055-bib-0036] Zhao, D. , Cai, L. , Wu, J. , Li, M. , Liu, H. , Han, J. , … Xiang, H. (2013). Improving polyhydroxyalkanoate production by knocking out the genes involved in exopolysaccharide biosynthesis in *Haloferax mediterranei* . Applied Microbiology and Biotechnology, 97, 36–3027. 10.1007/s00253-012-4415-3 23015099

